# Use of Oral Cholera Vaccines in an Outbreak in Vietnam: A Case Control Study

**DOI:** 10.1371/journal.pntd.0001006

**Published:** 2011-01-25

**Authors:** Dang Duc Anh, Anna Lena Lopez, Vu Dinh Thiem, Shannon L. Grahek, Tran Nhu Duong, Jin Kyung Park, Hye Jung Kwon, Michael Favorov, Nguyen Tran Hien, John D. Clemens

**Affiliations:** 1 National Institute of Hygiene and Epidemiology (NIHE), Hanoi, Vietnam; 2 International Vaccine Institute (IVI), Seoul, Republic of Korea; Massachusetts General Hospital, United States

## Abstract

**Background:**

Killed oral cholera vaccines (OCVs) are available but not used routinely for cholera control except in Vietnam, which produces its own vaccine. In 2007–2008, unprecedented cholera outbreaks occurred in the capital, Hanoi, prompting immunization in two districts. In an outbreak investigation, we assessed the effectiveness of killed OCV use after a cholera outbreak began.

**Methodology/Principal Findings:**

From 16 to 28 January 2008, vaccination campaigns with the Vietnamese killed OCV were held in two districts of Hanoi. No cholera cases were detected from 5 February to 4 March 2008, after which cases were again identified. Beginning 8 April 2008, residents of four districts of Hanoi admitted to one of five hospitals for acute diarrhea with onset after 5 March 2008 were recruited for a matched, hospital-based, case-control outbreak investigation. Cases were matched by hospital, admission date, district, gender, and age to controls admitted for non-diarrheal conditions. Subjects from the two vaccinated districts were evaluated to determine vaccine effectiveness. 54 case-control pairs from the vaccinated districts were included in the analysis. There were 8 (15%) and 16 (30%) vaccine recipients among cases and controls, respectively. The vaccine was 76% protective against cholera in this setting (95% CI 5% to 94%, *P* = 0.042) after adjusting for intake of dog meat or raw vegetables and not drinking boiled or bottled water most of the time.

**Conclusions/Significance:**

This is the first study to explore the effectiveness of the reactive use of killed OCVs during a cholera outbreak. Our findings suggest that killed OCVs may have a role in controlling cholera outbreaks.

## Author Summary

Simple measures such as adequate sanitation and clean water stops the spread of cholera; however, in areas where these are not available, cholera spreads quickly and may lead to death in a few hours if treatment is not initiated immediately. The use of life-saving rehydration therapy is the mainstay in cholera control, however, the rapidity of the disease and the limited access to appropriate healthcare units in far-flung areas together result in an unacceptable number of deaths. The WHO has recommended the use of oral cholera vaccines as a preventive measure against cholera outbreaks since 2001, but this was recently updated so that vaccine use may also be considered once a cholera outbreak has begun. The findings from this study suggest that reactive use of killed oral cholera vaccines provides protection against the disease and may be a potential tool in times of outbreaks. Further studies must be conducted to confirm these findings.

## Introduction

Cholera is increasingly being reported, and more countries are now experiencing outbreaks [1], some lasting for several months. In 2001, the World Health Organization (WHO) recommended the use of oral cholera vaccines (OCV) in populations at risk in endemic areas but not reactively once an outbreak has begun [2]. While this recommendation has been updated in March 2010, to include reactive use of these vaccines [3], OCVs have only been used for reactive cholera control in 2000, when a live attenuated OCV (CVD-103HgR) was used in an outbreak in Micronesia [4]. The CVD-103HgR was assessed to be effective in this outbreak, although this was an observational study. In contrast, CVD-103HgR conferred no protection in the only randomized controlled efficacy trial of this vaccine [5], and this vaccine is no longer manufactured. There is one internationally licensed killed oral cholera vaccine, the recombinant B subunit killed OCV (rBS-WC, Dukoral, Crucell/SBL), but it has not been routinely adopted for public health use due to its high cost, limited duration of protection and logistic issues with vaccine administration. A variant of this oral vaccine, containing only killed whole cells (*Vibrio cholerae* O1 and O139) is manufactured in Vietnam following technology transfer from Swedish scientists. Vietnam is the only country in the world to use an OCV in its public health system for cholera control. Since 1997, this killed OCV (ORC-Vax) has been licensed and produced locally by the Company for Vaccine and Biological Production (VaBiotech) in Hanoi. The vaccine was found to confer 66% protection against an El Tor cholera outbreak occurring eight months following vaccination among all individuals aged 1 year and older [6] and 50% protection, three to five years after vaccination [7]. It is safe, inexpensive, and easy to administer [8]. Packaged in five-dose vials, each 1.5 ml liquid vaccine dose is drawn and squirted into the mouth by a syringe without a needle. Each dose contained: 5.0×10^10^ formalin-killed *V. cholerae* Inaba, El Tor strain Phil 6973; 2.5×10^10^ heat-killed *V. cholerae* Ogawa, classical strain Cairo 50; 2.5×10^10^ formalin-killed *V. cholerae* Inaba, classical strain 569B; and 5.0×10^10^ formalin-killed *V. cholerae* O139 strain 4260B. After oral administration, individuals are asked to drink water, but no oral buffer is required. Given in two doses, one to four weeks apart, it may be given to individuals aged one year and older.

Since the seventh pandemic reached Vietnam in 1964, cholera has been reported annually. A review of reported cases to the National Institute of Hygiene and Epidemiology (NIHE) from 1991 to 2001 showed that cholera is endemic in the central and southern provinces [9]. Compared with shigellosis and typhoid fever, cholera cases have decreased dramatically in 1997 to 2001. This decrease in cholera cases has been partly attributed to the extensive use of the killed OCV in Vietnam [10].

**Figure 1:**
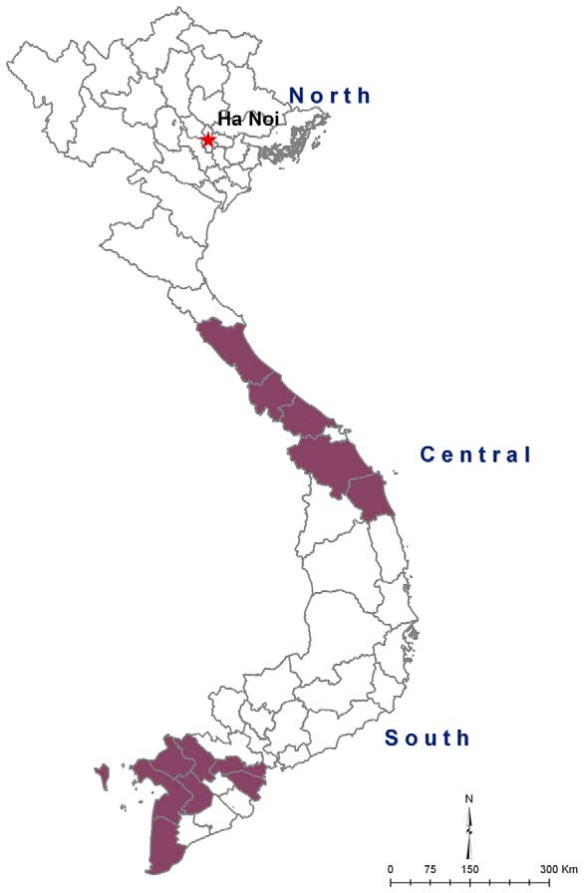
Map of Vietnam indicating cholera endemic areas in the Central coastal regions and in the South where cholera vaccines were used from 1997 to 2005.

From 1997 to 2005, 9.2 million doses of the killed OCV have been used in the Expanded Programme of Immunization (EPI) of 20 cholera endemic provinces and metropolitan areas in Vietnam, mostly located in the central and southern areas (Figure 1). Vaccines are routinely provided in the endemic areas through regular monthly immunization sessions. In the routine EPI setting, depending on the commune, eligible children, aged 2–5 years are gathered for immunization on the same days for cholera vaccination. OCVs are provided 2 to 4 weeks apart. The killed OCV is also used preemptively in mass campaigns whenever an increase in the number of culture-confirmed cases are reported. National diarrheal disease surveillance is performed routinely and culture confirmation of organisms is available at the 61 provincial Centers for Preventive Medicine and in the national and four regional Institutes of Hygiene and Epidemiology. When cholera cases are detected in known endemic areas, mass vaccinations are arranged in designated locations such as schools, commune and district health facilities or government offices in the affected areas.

In October 2007, an increase in acute watery diarrhea cases was reported in Hanoi, caused by genetically altered *Vibrio cholerae* O1 Ogawa biotype El Tor producing classical biotype cholera toxin. Prior to this outbreak, the strain had never been isolated in Vietnam [11]. From 24 October to 4 December 2007, nearly 2,000 diarrhea cases were reported from Hanoi and neighboring provinces, of which 295 were laboratory confirmed. In response the Ministry of Health of Vietnam mandated the provision of free medical treatment for anyone suffering from acute diarrheal illness.

New cholera cases were identified on 24 December 2007 from Hanoi, thus, in the first week of January 2008, just prior to the Vietnamese Tet New Year, a decision was made to immunize two particularly hard hit districts of Hanoi – Hoang Mai and Thanh Xuan (combined population of ∼462,570). These districts are located close to waterways into which sewage drains. The Vietnam National Institute of Hygiene and Epidemiology (NIHE) together with the Ministry of Health launched the mass vaccination campaign on 16–28 January 2008, providing two doses of the killed oral cholera vaccine, spaced one week apart. Because of the absence of cases detected during the outbreak among children less than 10 years of age, vaccines were only provided to residents aged 10 years and older. Pregnant residents were also not eligible for vaccination. The campaign was announced in newspapers and radio and eligible residents were invited to proceed to commune health centers. Vaccination cards were provided to vaccinees and logbooks containing the names of vaccine recipients were maintained. It was estimated that ∼80% of the estimated 370,000 age-eligible individuals received one or more doses of the killed OCV. In addition, educational health campaigns were also conducted to inform the public of the signs of illness and to improve sanitary practices.

**Figure 2:**
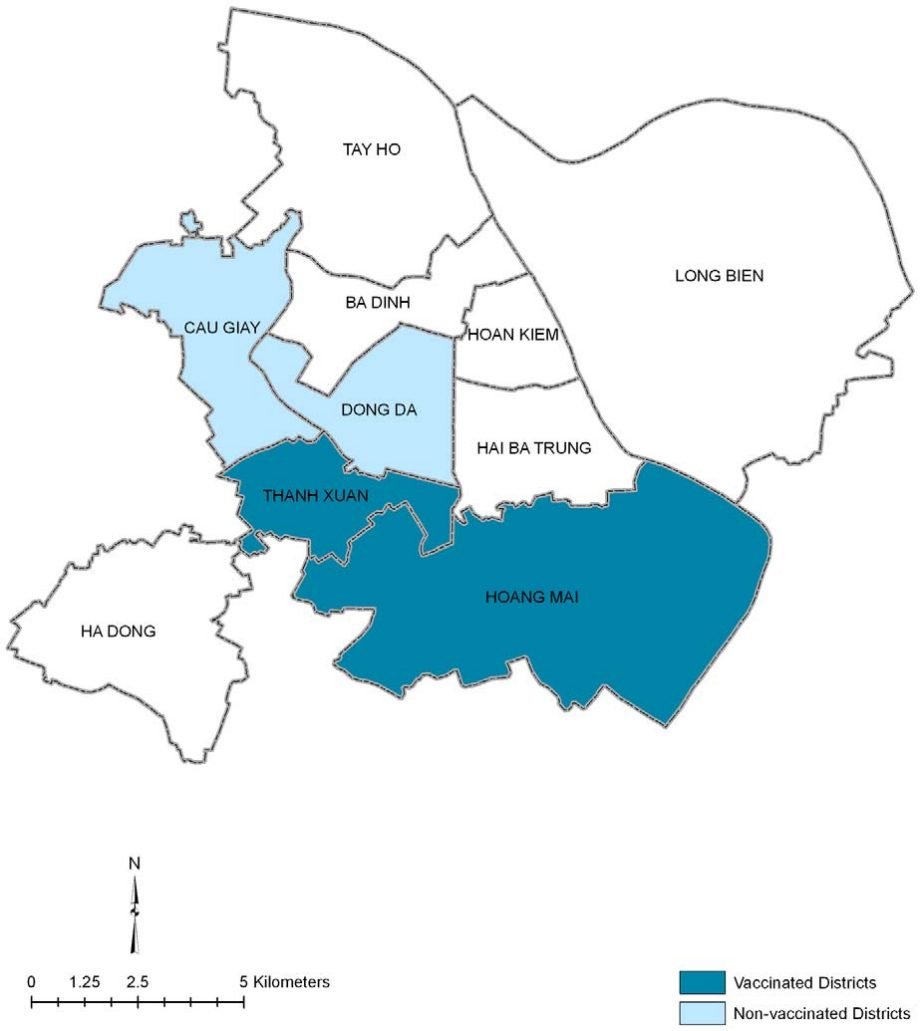
Clinical cholera cases in Hanoi, 2007 to 2008.

From 24 December 2007 to 6 February 2008, 59 diarrhea cases (33 culture confirmed *V. cholerae* O1) were identified, all cases coming from Hanoi. No cases were detected until 5 March 2008, when the number of diarrhea cases increased and *V. cholerae* O1 Ogawa was again identified as the causative agent. The NIHE requested the International Vaccine Institute (IVI) to assist in the outbreak investigation, specifically looking into the role of vaccines for control. This provided a unique opportunity to assess the effectiveness of reactive oral cholera vaccination in a cholera outbreak, as there has been little experience in the use of OCVs in cholera epidemics. Figure 2 shows the clinical cholera cases in Hanoi from 24 October 2007 to 15 July 2008.

## Methods

**Figure 3:**
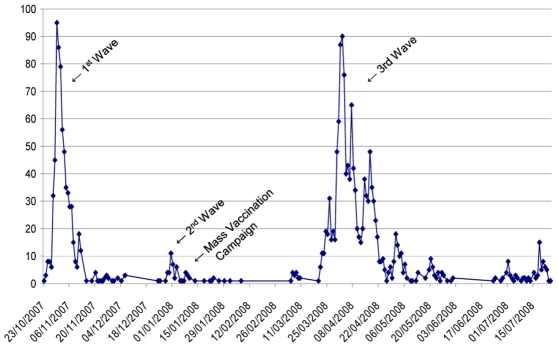
Urban districts of Hanoi showing Hoang Mai and Thanh Xuan, where mass vaccination campaigns were held in January 2008, and Dong Da and Cau Giay, the unvaccinated districts included in the study.

A matched, hospital-based, case-control investigation was conducted from 8 April to 10 June, 2008. Hanoi has nine urban districts with a population of ∼2.9 million [12]. Hospitalized patients from the two vaccinated districts - Hoang Mai and Thanh Xuan, as well as the unvaccinated districts - Dong Da and Cau Giay were invited to participate in the outbreak investigation (Figure 3). These districts have a combined population of ∼1 million [12]. These districts have similar population characteristics, environmental conditions and epidemiological data from past cholera outbreaks. Residents of these districts are also served in common and have equal chances of attending five hospitals including the National Institute of Infectious and Tropical Disease (NIID) Hospital, Bach Mai District Hospital, Saint Paul Hospital, Dong Da District Hospital and Transportation Hospital. Case and control exposure histories of subjects from Hoang Mai and Thanh Xuan , were compared for evaluation of risk factors and effectiveness of killed OCV use during the outbreak, the results of which are presented here.

### Participants: Selection of Cases

Patient admission logbooks at the five hospitals were reviewed daily to identify patients admitted for diarrhea. Hospital records of identified patients were then reviewed. Patients who met the clinical case definition for cholera were invited to participate. A cholera case was defined, a priori, as being hospitalized for diarrhea with illness onset of 8 April to 20 May 2008, with diarrhea defined as 3 or more loose, liquid or watery bowel movements in any 24 hour period; were 10 years of age or older and a resident of any of the 4 districts of interest. Cases were identified without knowledge of the vaccination status.

### Participants: Selection of Controls

One matched control per case was recruited from wards of the same hospital, except for cases admitted to NIID, wherein controls were identified from the trauma and surgical wards of Bach Mai Hospital, an adjacent general hospital. Patient admission logbooks were reviewed to identify controls hospitalized for non-diarrheal conditions. Controls were matched for each case by the date of presentation (±5 days), age group (10–20 years old, 21–40 years old, 40+ years old), gender and district of residence. The first control in the logbook that fulfilled the matching characteristics to the case was identified and invited to participate. Controls were chosen by reviewers who were unaware of the vaccination status of the patients.

### Data Collection Procedures

Data were obtained through transcription of clinical records and subject interviews using a standardized questionnaire. Demographic characteristics including occupation, water supply (tap water, public well), behavioral characteristic such as hand washing and sanitation (toilet with flush, latrine, none), as well as exposure factors (intake of raw vegetables, dog meat, shrimp paste; not drinking boiled or bottled water), were collected. Vaccination status including the number and date of dosing was verbally ascertained based on subject recall. When available the reported dosing dates were cross-checked against a vaccination card. In order to evaluate the use of the OCV in this outbreak setting we defined “vaccinated” a priori as receipt of one or two doses of OCV from 16–28 January 2008 without further consideration to dosing interval or interval between vaccination and date of selection into the study. Microbiological culture results, completed by and according to the standard operating procedures of the admitting hospital laboratory, were also obtained during the study when available.

### Statistical Methods

To detect 50% vaccine protection, we assumed the following: 40% of controls would be vaccinated; the correlation of vaccine histories among matched cases and controls, phi, was .05; and with 80% power at P<.05 (2-tailed), at least 172 cases and 172 controls were required for the investigation.

Characteristics and exposures of hospitalized cases and controls from the vaccinated and unvaccinated districts were compared. To assess the effect of vaccination, we included diarrheal cases and controls hospitalized for non-diarrheal causes from the vaccinated districts. Baseline characteristics were statistically compared using McNemar's test for dichotomous variables and the paired Student t-test for continuous variables. Only complete pairs in which both the case and the control had exposure measurements were included, and the information contained in the incomplete pairs was ignored. The adjusted matched odds ratio (OR) and 95% confidence interval (CI) for calculation of vaccine effectiveness was determined using multivariate conditional logistic regression [13]. Statistical analysis was planned at the outset, to include all variables with *p*<0.05 in univariate analysis and the primary variable of interest (vaccination status) in the multivariable model. Vaccine effectiveness was calculated as: (1-matched OR)×100. All p values and 95% confidence intervals, estimated from the point estimates and standard errors for the coefficient for the vaccination variable in the models, were interpreted in a two- tailed manner. Statistical significance was designated as a p value<0.05. All statistical analyses were performed using Stata10 (StataCorp, College Station, TX).

### Ethics

The study qualified for exemption from review by the IVI Institutional Review Board and Ethical Review Committee of NIHE as the study was conducted as part of an outbreak investigation establishing risk factors and modifiers. Verbal consent was obtained in lieu of written consent from both cases and controls as the project was conducted as part of an outbreak investigation. Consent was documented in a logbook.

## Results

**Figure 4:**
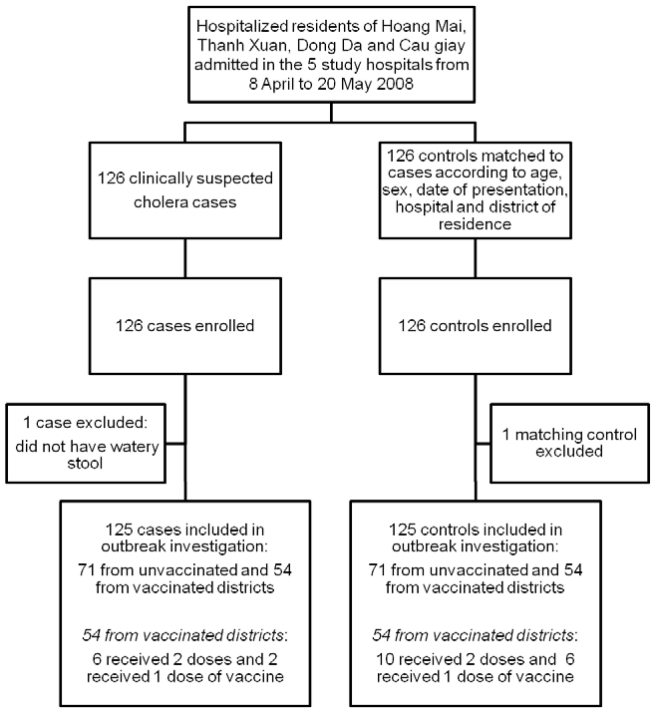
Flowchart of cases and controls in the study.

We enrolled 126 matched pairs of cases and controls for the outbreak investigation; one matched pair was excluded when on review the case definition was not met by the case (Figure 4). After exclusion of this matched pair, among cases, the ages ranged from 17 to 86 years old while the control age range was 15 to 80 years old. Thirty-seven percent of cases had vomiting and 76% had some or severe dehydration on admission. Among those with severe dehydration, only one was vaccinated. Of the 99 cases whose stools were tested, 74 subjects had culture confirmed *V. cholerae* O1 (75%). Only one vaccine recipient had culture confirmed cholera. Table 1 shows the causes of hospitalization for the controls.

**Table 1:** Cause of hospitalization among controls.

Cause of hospitalization*	No. of controls (n = 125)
Respiratory	32
Dental Problem	21
Dengue, viral or unspecified febrile disease	19
Gastrointestinal	10
Neurological	10
Cardiovascular	9
Urogenital	5
Endocrine	3
Musculoskeletal Trauma	3
Skin / Soft Tissue Problem	2
Other†	11

*No controls were admitted for or experienced diarrhea during hospitalization.

**†:** Alcoholism, cancer, unspecified non-infectious condition.

Of the 125 matched pairs, 54 pairs (43%) were residents of districts where the mass vaccination campaign was carried out and were included in this evaluation of vaccine effectiveness. We compared the baseline characteristics of cases and controls from Huang Mai and Thanh Xuan, where the mass vaccination campaigns were carried out, and found no significant differences in demographic and socio-economic characteristics (Table 2). On comparing the exposure of cases with controls, intake of raw vegetables and not drinking boiled or bottled water were found to be significantly different (p<0.05). Similar results were obtained when comparing all cases and controls in the outbreak investigation, including patients from both the vaccinated and unvaccinated districts (data not shown). Because dog meat is customarily eaten with raw vegetables and 70% of those who ate dog meat also ate raw vegetables, we decided to combine these in the multivariate regression model.

**Table 2:** Characteristics of diarrheal cases compared with controls hospitalized for other conditions during a cholera outbreak in Hanoi, Vietnam, 2008 (vaccinated districts).

Variable	Discordant pairs (case exposed)	Unadjusted matched odds ratio	p-value
Mean age (years)* †		1.00	0.897
Mean monthly income (USD)*		1.00	0.648
Male gender†	3 (1)	2.00	0.571
Occupation requiring frequent travel‡	12 (9)	3.00	0.099
Ate dog meat§	13 (10)	3.33	0.067
Ate raw vegetables∥	18 (14)	3.50	0.027
Ate dog meat or raw vegetables¶	21 (17)	4.25	0.009
Ate shrimp paste**	11 (6)	1.200	0.763
Do not drink boiled or bottled water most of the time††	12 (11)	11.000	0.022
Live in household without safe water/household tap‡‡	6 (2)	0.500	0.423

*Modeled as continuous variable.

**†:** Matching factor.

**‡:** Examples of occupations requiring little or no travel include homemakers, seamstresses and office workers, while examples of occupations requiring frequent travel include laborers, car drivers and tour guides. Information available for 50 pairs, no missing information among discordant pairs.

**§:** Information available for 53 pairs, no missing information among discordant pairs.

**∥:** Information available for 53 pairs, no missing information among discordant pairs.

**¶:** Information available for 53 pairs, no missing information among discordant pairs.

**: Information available for 53 pairs, no missing information among discordant pairs.

**††:** Information available for 52 pairs, no missing information among discordant pairs.

**‡‡:** Information available for 52 pairs, no missing information among discordant pairs.

Of subjects from the vaccinated districts, 8 of 54 cases (15%) and 16 of 54 controls (30%) were classified as vaccinated, having received at least one dose of the killed OCV from 16–28 January 2008. Seventy-five percent (6/8) of vaccinated cases and 63% of vaccinated controls (10/16) received two doses of killed OCV during the vaccination campaign. The unadjusted vaccine effectiveness (VE) was 54% (95% CI −31% to 84%; p-value = 0.144), however, after adjusting for factors which were found to be significantly associated with being a cholera case at P<0.05 in univariate analyses (intake of dog meat or raw vegetables and not drinking boiled or bottled water most of the time) (Table 3), the killed OCV was found to have an effectiveness of 76% (95% CI 5% to 94%, p = 0.04).

**Table 3:** Vaccine effectiveness against clinical cholera in Huang Mai and Thanh Xuan following the mass vaccination campaign in 2008 (vaccinated districts).

		Controls		
		Vaccinated	Unvaccinated	Total
Cases	Vaccinated	3	5	8
	Unvaccinated	11	35	46
	Total	14	40	54

Vaccinated was defined as having received one or two doses of vaccine.

Crude Protective Efficacy (PE) = 54% (95% CI −31% to 84%; p-value = 0.144).

Adjusted PE = 76% (95% CI 5% to 94%; p-value = 0.042).

Stepwise selection (P>0.05 for removal), candidate variables vaccination status plus variables significant (P≤0.05) in univariate analysis; dog meat or raw vegetables, and not drinking bottled or boiled water most of the time.

## Discussion

This is the first study to report on the use of killed OCV in an outbreak situation. While a significant association was detected between receipt of at least one dose of the killed OCV and protection against cholera, our study has several limitations.

### Potential Limitations

Because there may be inherent differences in health care utilization and knowledge among those who presented for vaccination and those who refused vaccination [14], [15], bias may have been introduced in our assessment for vaccine protection, and may have exaggerated our results. The protective effect may have been augmented, as it has been shown that people refusing participation are more likely to engage in high-risk behaviors as compared to vaccines [15]. However, there were no differences in the baseline demographic, socioeconomic and exposure characteristics of vaccinated and non-vaccinated cases and controls. Moreover, there were several factors that may have decreased the true protective effect of the vaccine during this outbreak, namely: (1) individuals with a recent history of cholera-like diarrhea may not have participated in the campaign and were included in the control group (2) recipients of a single dose of the vaccine were included in the analysis (3) vaccinees may have been more likely to use the treatment centers for the care of diarrhea compared to refusers. A comparison of a partially immunized vaccine group to a control group with varying levels of natural immunity would tend to depress apparent vaccine protection against subsequent cholera.

Our evaluation was also limited by use of a clinical case definition without culture confirmation, however we used a strict case definition and random cases were culture confirmed. Moreover, inclusion of non-culture confirmed cases, if ever, would have depressed the protection afforded by vaccination as some cases may not be due to *V. cholerae*.

We did not reach the sample size required (54 instead of the desired 172) because of difficulty enrolling controls during this outbreak, throughout which most hospital beds were occupied by cholera cases. The smaller sample size may explain the unadjusted VE as being not statistically significant. We tried to limit selection bias by enrolling cases and controls without prior knowledge of their vaccination status. Moreover, in order to prevent interviewers from overzealously eliciting vaccination history, several exposure questions were included in the questionnaire.

Lastly, our study was initiated more than two months after the campaign, thus we were unable to include cases proximate to vaccination, however since the outbreak was prolonged and recurrent and vaccine effectiveness lasts for three to five years [7], measurement of the effectiveness of OCV use in this setting was still warranted.

### Implications for the Control of Cholera

To our knowledge, this is the first study that explored the reactive use of a killed OCV in an outbreak. In Hanoi, the outbreak was described as having occurred in three waves, each separated by 14 to 26 day intervals with no recorded cases in between each wave. Vaccination was performed while the second wave was ongoing (see Figure 3). Since the mass vaccination campaign was performed in the two districts that have been most affected in the previous waves of diarrheal cases, the characteristics of the residents in these districts may have been different from other areas that make them vulnerable to diarrheal outbreaks and may be more amenable to district specific interventions. However, comparison of baseline characteristics and exposures of patients from the vaccinated (Hoang Mai and Thanh Xuan) and unvaccinated districts (Dong Da and Cau Giay) showed no statistically significant differences (data not shown).

In the recently updated WHO recommendations, consideration for both preemptive and reactive use of OCVs is supported after assessment of local infrastructure and epidemiology. A model of a refugee camp based cholera outbreak in Africa compared the cost-effectiveness of several cholera controls strategies, including establishment of treatment centers and reactive vaccination. Based on duration of the hypothetical outbreak and the size of the hypothetical camp, reactive vaccination will only be a cost-effective option if the price of the vaccine falls below $0.22 per dose [16]. However, there were several limitations to this analysis [17] and this study did not account for large prolonged outbreaks such as those seen recently in Zimbabwe [18], [19], Angola [20], [21] and Vietnam [11], which would favor reactive vaccination.

Since 1996, extensive cholera outbreaks of this magnitude had not been reported in Vietnam, especially in areas where the killed OCV is routinely used. Between 5 March and 22 April 2008, the Vietnamese Ministry of Health reported 2,490 cases of severe acute watery diarrhea including 377 that were positive for *V. cholerae* O1 Ogawa [22]. Twenty provinces in the northern areas were affected in 2007 to 2008. No deaths were reported during these outbreaks indicating good case management. On the other hand, in Africa, cholera outbreaks are deadly. In Zimbabwe alone from August 2008 to May 2009, almost 100,000 cases have been identified with more than 4,000 deaths [18], 61% of whom did not reach a health facility for treatment [19]. Similarly in Angola, an outbreak from February to June 2006 with 46,758 cases and 1,893 deaths [20], [21] were reported with case fatality rates in some provinces tragically reaching up to 30% [20]. Provisions for clean water, adequate sanitation and good case management are necessary for controlling cholera, however, these are unlikely to happen in the near future in most of the developing world where cholera continues to cause significant hardship and misery. New measures need to be taken. Prior to the release of the March 2010 WHO position paper several groups were pressing for a rethink of the WHO stand on vaccine use for outbreaks [23]. The results of our study are consistent with earlier evaluations of the protective effects of OCV [6]. Microbiologic studies have shown that the outbreak was caused by the new strain of El Tor *V. cholerae* O1 producing classical cholera toxin [11]. This new strain has been increasingly reported in Asia and in parts of Africa [24]–[26] with some indications of increased severity [27]. The killed OCV provided protection against this new strain suggesting that there may be a role for reactive use of the killed OCV in future cholera outbreaks.

The Vietnamese killed OCV has now been extensively modified by the IVI to comply with WHO and current Good Manufacturing Practices (cGMP) standards. The modified vaccine was recently licensed in Vietnam (mORC-VAX). In order to expand its use internationally and to allow purchase by United Nations agencies, technology transfer of the vaccine production process was made by the IVI to Shantha Biotechnics in India where it is now licensed (Shanchol®). This modified vaccine with higher antigenic content than the previous versions has been found to be safe and protective in India [28] and resulted in comparable vibriocidal immune responses after one or two doses of the vaccine raising the possibility that it may be used as a single dose, which would greatly simplify vaccine delivery in times of outbreaks [29]. Further studies to confirm our findings are necessary; however, these results provide hope that the vaccine will be used not only for endemic cholera control but in times of outbreaks as well, when mortality may be higher [30].

## Supporting Information

Checklist S1 STROBE checklist.
(DOC)
